# Evaluation of Myocardial Gene Expression Profiling for Superior Diagnosis of Idiopathic Giant-Cell Myocarditis and Clinical Feasibility in a Large Cohort of Patients with Acute Cardiac Decompensation

**DOI:** 10.3390/jcm9092689

**Published:** 2020-08-19

**Authors:** Felicitas Escher, Heiko Pietsch, Ganna Aleshcheva, Philip Wenzel, Friedrich Fruhwald, Christian Stumpf, Dirk Westermann, Johann Bauersachs, Frank Enseleit, Frank Ruschitzka, Herbert Nägele, Karl-Ludwig Laugwitz, Hendrik Haake, Norbert Frey, Johannes Brachmann, Kurt Huber, Rüdiger Christian Braun-Dullaeus, Martin W. Bergmann, Jörg Strotmann, Gerian Grönefeld, Jürgen Krülls-Münch, Ralf Westenfeld, Carsten Skurk, Ulf Landmesser, Burkert Pieske, Ulrich M. Gross, Lars Morawietz, Heinz-Peter Schultheiss

**Affiliations:** 1 Institute of Cardiac Diagnostics and Therapy, IKDT GmbH, 12203 Berlin, Germany; heiko.pietsch@ikdt.de (H.P.); info@ikdt.de (G.A.); ugross@zedat.fu-berlin.de (U.M.G.); l.morawietz@mvz-fuerstenberg-karree.de (L.M.); heinz-peter.schultheiss@ikdt.de (H.-P.S.); 2 Department of Cardiology, Charité–University Medicine Berlin, Campus Virchow-Klinikum, 13353 Berlin, Germany; burkert.pieske@charite.de; 3 German Centre for Cardiovascular Research (DZHK), Partner Site Berlin, Berlin, Germany; carsten.skurk@charite.de (C.S.); Ulf.Landmesser@charite.de (U.L.); 4 Department of Cardiology and Center for Thrombosis and Haemostasis, University Medical Center Mainz, 55131 Mainz, Germany; wenzelp@uni-mainz.de; 5 Department of Internal Medicine, Division of Cardiology, Medical University Graz, 8036 Graz, Austria; friedrich.fruhwald@medunigraz.at; 6 Department of Cardiology, Klinikum Bayreuth GmbH, Medical Clinic II, 95445 Bayreuth, Germany; christian.stumpf@klinikum-bayreuth.de; 7 Department of Internal Medicine and Interventional Cardiology, University Heart Center Hamburg, 20251 Hamburg, Germany; d.westermann@uke.de; 8 Department of Cardiology and Angiology, Hannover Medical School, 30625 Hannover, Germany; bauersachs.johann@mh-hannover.de; 9 Department of Cardiology, University Hospital Zurich, 8091 Zurich, Switzerland; frank.enseleit@usz.ch (F.E.); frank.ruschitzka@usz.ch (F.R.); 10 Department for Cardiac Insufficiency and Device Therapy, Albertinen-Hospital, 22457 Hamburg, Germany; herbert.naegele@albertinen.de; 11 Department of Cardiology, Klinikum rechts der Isar, 81675 Munich, Germany; laugwitz@mytum.de; 12 Department of Cardiology, Electrophysiology and Intensive Care Medicine, Kliniken Maria Hilf GmbH, 41063 Monchengladbach, Germany; Hendrik.Haake@mariahilf.de; 13 Department of Internal Medicine III-Cardiology, Angiology and Intensive Care Medicine, University Hospital Schleswig-Holstein, 24105 Kiel, Germany; Nobert.Frey@ukhs.de; 14 Department of Internal Medicine, Division of Cardiology, Clinical Center Coburg, 96450 Coburg, Germany; johannes.brachmann@klinikum-coburg.de; 15 3rd Medical Department with Cardiology, Wilhelminenhospital Vienna, 1160 Vienna, Austria; kurt.huber@wienkav.at; 16 Department of Cardiology und Angiology, University Magdeburg, 39106 Magdeburg, Germany; r.braun-dullaeus@med.ovgu.de; 17 Cardiologicum Hamburg Wandsbek, 22041 Hamburg, Germany; m.bergmann@cardiologicum.net; 18 Department of Cardiology, Städtisches Krankenhaus Kiel GmbH, 24116 Kiel, Germany; Joerg.strotmann@krankenhaus-kiel.de; 19 Department of Cardiology, Asklepios Klinik Barmbek, 22307 Hamburg, Germany; g.groenfeld@asklepios.com; 20 Department of Cardiology and Angiology, Carl-Thiem-Klinikum Cottbus gGmbH, 03048 Cottbus, Germany; 1.Med.Klinik@ctk.de; 21 Division of Cardiology, Pulmonology and Vascular Medicine, Medical Faculty, Heinrich-Heine University, 40225 Düsseldorf, Germany; Ralf.Westenfeld@med.uni-duesseldorf.de; 22 Department of Cardiology, Charité–University Medicine Berlin, Campus Benjamin Franklin, 12203 Berlin, Germany

**Keywords:** idiopathic giant-cell myocarditis, gene-expression profiling, endomyocardial biopsy

## Abstract

*Aims*: The diagnostic approach to idiopathic giant-cell myocarditis (IGCM) is based on identifying various patterns of inflammatory cell infiltration and multinucleated giant cells (GCs) in histologic sections taken from endomyocardial biopsies (EMBs). The sampling error for detecting focally located GCs by histopathology is high, however. The aim of this study was to demonstrate the feasibility of gene profiling as a new diagnostic method in clinical practice, namely in a large cohort of patients suffering from acute cardiac decompensation. *Methods and Results*: In this retrospective multicenter study, EMBs taken from *n* = 427 patients with clinically acute cardiac decompensation and suspected acute myocarditis were screened (mean age: 47.03 ± 15.69 years). In each patient, the EMBs were analyzed on the basis of histology, immunohistology, molecular virology, and gene-expression profiling. Out of the total of *n* = 427 patient samples examined, GCs could be detected in 26 cases (6.1%) by histology. An established myocardial gene profile consisting of 27 genes was revealed; this was narrowed down to a specified profile of five genes (*CPT1, CCL20, CCR5, CCR6, TLR8*) which serve to identify histologically proven IGCM with high specificity in 25 of the 26 patients (96.2%). Once this newly established profiling approach was applied to the remaining patient samples, an additional *n* = 31 patients (7.3%) could be identified as having IGCM without any histologic proof of myocardial GCs. In a subgroup analysis, patients diagnosed with IGCM using this gene profiling respond in a similar fashion to immunosuppressive therapy as patients diagnosed with IGCM by conventional histology alone. *Conclusions*: Myocardial gene-expression profiling is a promising new method in clinical practice, one which can predict IGCM even in the absence of any direct histologic proof of GCs in EMB sections. Gene profiling is of great clinical relevance in terms of (a) overcoming the sampling error associated with purely histologic examinations and (b) monitoring the effectiveness of therapy.

## 1. Introduction

Cardiac inflammatory processes involving giant cells comprise a diverse group of disorders [1,2,3,4] Idiopathic giant-cell myocarditis (IGCM) is regarded as a distinct clinical and pathological entity having an exclusively cardiac manifestation. This rapidly progressive disease is associated with myocyte necrosis and poor cardiac outcome [5,6]. IGCM has been shown to involve multinucleated giant cells (GCs) which have thus far been discovered mainly in lymphocytic infiltrates and among myocytolytic tissue and eosinophils [7,8,9]. Since GCs tend to be focally distributed within endomyocardial biopsies (EMBs), their presence is very often missed by conventional histologic evaluation due to the sampling error involved. Thus, the actual incidence of IGCM could well be higher than its detection rate. The earlier IGCM is diagnosed and immunosuppressive treatment is initiated, the better the patient recovers, given that permanent myocardial damage can be prevented (or at least minimized), thereby improving the prognosis and possibly avoiding the need for heart transplantation. Recently published studies have shown [10,11] that myocardial gene-expression profiling defines a distinct gene expression pattern which serves to indicate the presence of IGCM even without any histologic detection of GCs. Generally speaking, specific gene-expression profiles describe the time-specific and disease-specific synthesis of cytokines and adhesion molecules, thereby defining the activation states of pro-inflammatory and anti-inflammatory intracellular pathways [12,13,14]. Thus, gene-expression profiling has recently been assumed to play an increasingly important diagnostic role for rejection surveillance after cardiac transplantations [15,16,17]. Given the focal infiltration pattern which GCs exhibit in cardiac tissue, novel methods for diagnosing IGCM are urgently needed. This multicenter study addresses the clinical evaluation of gene profiling for purposes of identifying patients afflicted with IGCM. The aim of this study was (a) the identification of a distinct gene-profiling, (b) to demonstrate the feasibility of gene profiling in clinical practice within a large cohort of patients, and (c) to show the efficacy of subsequently applied immunosuppressive treatment regarding the prognosis of the disease.

## 2. Patients and Methods

### 2.1. Patients

This retrospective multicenter study evaluated the EMB specimens of *n* = 427 patients suffering from clinically unexplained acute decompensation; these specimens had been sent to the IKDT (Institute for Cardiac Diagnostic and Therapy Berlin, Germany). Analysis included histology, immunohistochemistry, molecular virology, and gene profiling. The suspected clinical diagnoses had been made by clinicians at the relevant medical centers. In order to develop gene-expression profile that could serve as a novel tool for the diagnosis of GCs, twenty-three age-matched and gender-matched patients without intramyocardial inflammation or viral infection were used as a peer group to create the control-group profiles. They were referred for evaluation of repeated chest discomfort but had no symptoms of heart failure. Patient characteristic and hemodynamic data are summarized in Table 1 (see also Supplemental Data Table S1 for EMB results). 

### 2.2. Analysis of Myocardial Morphology and Inflammation

Histologic evaluations were performed on paraffin sections of two to three EMBs using standard procedures, e.g., formaldehyde or RNA*later* fixation, paraffin embedding, staining with hematoxylin and eosin, elastic van Gieson stain (EvG) and Azan stain. The EMB diagnosis of active myocarditis was based on the histomorphologic criteria according to the Dallas Classification [18]. Immunohistochemistry was used to characterize the inflammatory infiltrates and was carried out on RNA*later*-fixed samples (two EMBs). Myocardial inflammation was diagnosed by CD3^+^ T-lymphocytes/mm^2^ (Dako, Glostrup, Denmark), CD11a^+^/LFA-1^+^ lymphocytes/mm^2^ (Immuno Tools, Friesoythe, Germany), CD11b^+^/Mac-1^+^ macrophages/mm^2^ (ImmunoTools), CD45R0^+^ T memory cells (Dako, Glostrup, Denmark), perforin^+^ cytotoxic cells/mm^2^ (BD Bioscience, San Jose, CA, USA). Inflammatory cells were quantified using quantitative digital-imaging analysis, reported elsewhere [19]. Intramyocardial inflammation was categorized according to the *ESC* Statement [20]. We also analysed macrophages (threshold >40.0 CD11b^+^/Mac-1^+^ macrophages/mm^2^), CD45R0^+^ T Memory cells (threshold > 40 cells/mm^2^), and perforin-positive cytotoxic cells (threshold > 2.9 cells/mm^2^). 

### 2.3. Nucleic Acid Isolation, Reverse Transcription (RT) and nPCR for cDNA

Genomic DNA from two to three EMBs was extracted using Puregene Core Kit A (Qiagen, Hilden, Germany). Total RNAs were isolated during routine EMB diagnostics using Trizol reagent (Thermo Fisher Scientific, Waltham, MA, USA); these were treated with DNAse (PeqLab, Erlangen, Germany) to remove any traces of genomic DNA and were then reverse-transcribed to cDNA with the High Capacity Kit (Thermo Fisher Scientific) using random hexamer primers. DNA and cDNA concentrations were quantified using the PCR-based Quantifiler™ Human DNA Quantification Kit (Thermo Fisher Scientific).

### 2.4. Preamplification and Gene-Expression Analysis

Given the limited amounts of extracted myocardial cDNA available, a gene-specific PCR-based pre-amplification technique was applied. Gene expression was then determined via qPCR amplification of generated preAMP-DNA. The expression level was calculated in relation to the housekeeping gene *HPRT* while applying the Delta-Delta-Ct method [21]. All predesigned gene-expression qPCR assays were purchased from Thermo Fisher Scientific and applied in keeping with the manufacturer’s instructions. To ensure the technical integrity of the gene profile test, samples that produced low amounts of RNA were excluded from further analysis. The cut-off values were set at Ct < 25 for *HPRT* detection and for Quantifiler^TM^ analysis, respectively. Samples of each patient were run in parallel with *HPRT* for quantification of mRNA and internal assay amplification control to ensure standardization of PCR. Previous *in vivo* and *in vitro* microarray-based studies had identified a set of 27 genes that have been shown to be deregulated by inflammatory cardiomyopathy. These genes serve as coding for cellular receptors or immune-response mediators, or are part of energy metabolism pathways [11] (Table S2). The evaluation of the myocardial gene-expression profiles revealed a specific expression pattern encompassing five specific genes: chemokine receptor 5 (*CCR5*), chemokine receptor 6 (*CCR6*); carnitine palmitoyltransferase I (*CPT1*), toll-like receptor 8 (*TLR8*), and chemokine (C-C motif) ligand 20 (*CCL20*) [22,23,24,25,26,27]. Expression of *CPT1* was found to be downregulated in IGCM.

### 2.5. Statistics

The quantitative results of the analysis were expressed as mean ± SD (standard deviation) values. The parametric paired *t*-test was used to analyze data within a group, whereas the parametric unpaired *t*-test was used to compare different groups. Once it had been established that none of the data were distributed normally, the non-parametric Mann-Whitney U test for group comparisons and Wilcoxon’s signed rank test for comparisons between baseline and follow-up were utilized. The non-parametric Spearman correlation method was used for correlation analysis. *P*-values below 0.05 were treated as indicators of statistical significance. All statistical analyses were performed using Version 23.0 of the SPSS software, (IBM Corp. Armonk, NY, USA), as well as the GraphPad Prism 7.04 software (GraphPad Software Inc., La Jolla, CA, USA).

## 3. Results

The patients included in this study were evaluated by means of extensive EMB analysis, including histologic, immunohistochemical, molecular virology analyses and gene profiling. A summary overview of the patients’ characteristics, hemodynamic data and suspected clinical diagnoses can be found in Table 2.

In *n* = 26 patients, the incidence of IGCM could be determined through histopathologic analyses of EMBs. All the patients included in this study were considered for purposes of establishing the specific gene-expression profile.

Histology-proven IGCM was found to be present in *n* = 26 patients. In this patient group, *n* = 23 patients fulfilled the criteria for active myocarditis according to the Dallas Classification; in the remaining *n* = 3 patients, borderline cases of myocarditis were indicated. 

**Figure 1:**
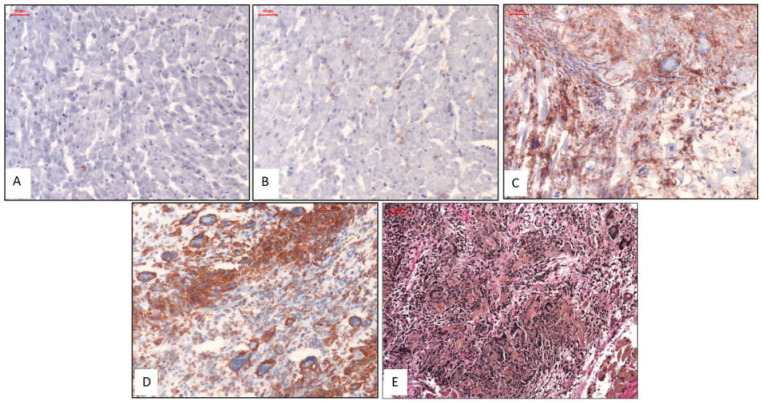
Representative Histologic and Immunohistochemical Images. Images (**A**) & (**B**): Patients with normal myocardium; i.e., no CD3 stain (**A**) and no Mac1 stain (**B**). Images (**C**) & (**D**): Patients with IGCM presenting GCs surrounded by diffuse infiltration of massively increased T lymphocytes (CD3) (**C**) and macrophages (Mac1) in immunohistologic staining (**D**). Image (**E**): EvG staining from a patient with severe active myocarditis and giant cells. Magnification ×200.

Representative images of the histologic and immunohistologic findings are shown in Figure 1. 

### 3.1. Distinctive Myocardial Gene-Expression Profiles Which Serve to Identify IGCM

**Figure 2:**
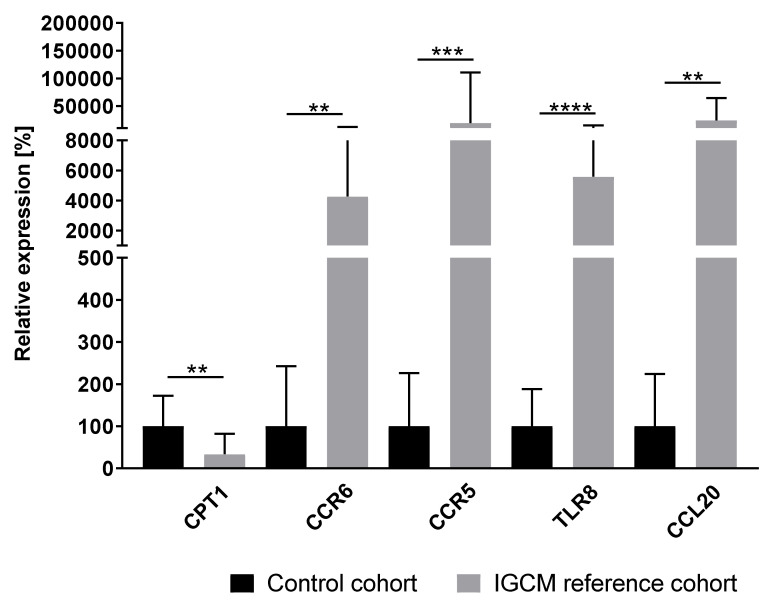
Gene-expression profiles for IGCM patients (*n* = 26) in relation to inflammation-negative patients/control cohort (*n* = 23). The figure shows a distinct gene-expression pattern with high statistical significance, as derived from the unpaired *t*-test. *P*-values are denoted by asterisks: ** *p* ≤ 0.01, *** *p* ≤ 0.001, **** *p* ≤ 0.0001.

EMBs taken from the 26 patients with histologically confirmed IGCM were analyzed. The gene-expression profile could be used to successfully identify histologically proven IGCM with a high degree of specificity in 25 of the 26 patients (96.2%) (Figure 2, Figure S1, Table S3). 

### 3.2. Application of IGCM-Specific Gene Profiling across the Entire Cohort of Patients with Acute Cardiac Decompensation

As a next step, the gene-expression data of the remaining patients (*n* = 401) who exhibited acute cardiac decompensation but no histologically detected GCs were evaluated based on the new numeric thresholds of the five genes identified for the IGCM-specific gene profile. When this newly derived profiling was applied, an additional *n* = 31 patients could be identified as having a distinctive gene-expression pattern suggestive of IGCM; these patients were therefore classified as suspected cases of IGCM despite the absence of histologic proof for the presence of myocardial GCs. 

**Figure 3:**
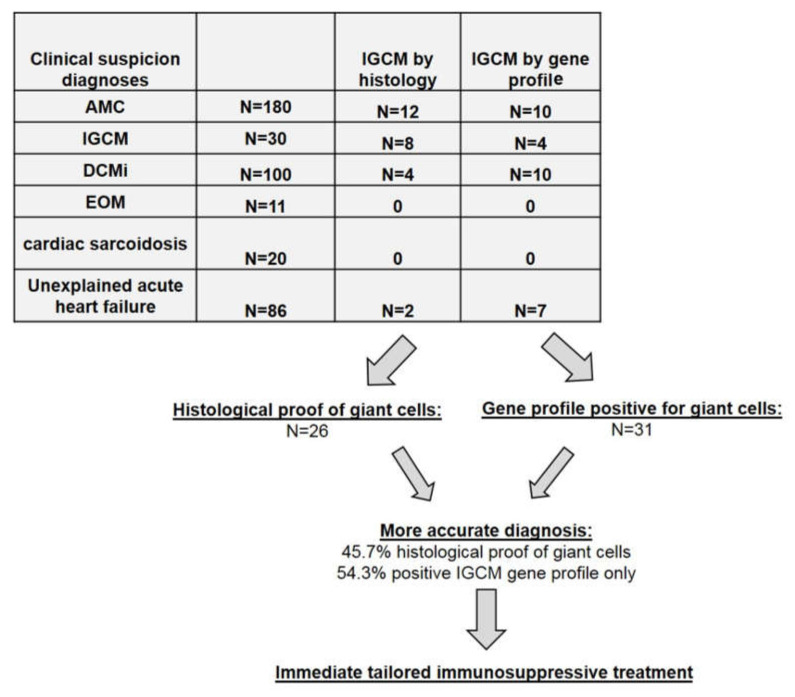
Clinically Suspected Diagnoses and IGCM Histologically Proven or Detected by Gene-Profiling. Note: AMC = acute myocarditis; IGCM = idiopathic giant cell myocarditis; DCMi = dilated inflammatory cardiomyopathy; EOM = eosinophilic myocarditis.

In particular, a gene profile matching the criteria for multinucleated giant cells could be observed in *n* = 14 of the patients with clinically suspected acute myocarditis. In *n* = 10 of the patients presenting a positive gene profile for GCs, an inflammatory cardiomyopathy had been clinically suspected; in *n* = 7 patients, unexplained acute heart failure had been diagnosed (Figure 3). Out of these 31 patients, an evaluation of EMBs determined that *n* = 6 patients actually had active myocarditis according to the Dallas Classification based on histology. In the remaining *n* = 25 patients, borderline myocarditis was identified (Figure 3).

Including samples taken from the 427 patients suffering from acute cardiac decompensation, multinucleated giant cells were ultimately detected in a total of 26 (6.1%) of the patients by means of histology. However, based on the improved diagnostics offered by myocardial gene profiling, an additional *n* = 31 patients of the remaining cohort could also be identified as having giant cells even without any direct histologic proof of such cells. Thus, out of the overall cohort of 427 patients with acute cardiac decompensation, a total of 57 patients (13.3%) could be diagnosed as having IGCM. Only 26 of these 57 (45.6%) had a histologic presentation of GCs. In other words, *n* = 31 of those 57 (54.4%) would have been overlooked if conventional histology alone had been used. 

### 3.3. Immunohistologic Analysis of Intramyocardial Infiltration in GCM Patients

When it comes to the immunohistologic staining found among the IGCM patient samples, the number of infiltrative cells involved ranges widely. See Table 3 for a breakdown of IGCM diagnoses based on conventional, histologic proof and those based on gene profiling.

### 3.4. Correlation of Immunohistochemical Markers with Deregulated Genes

In the total patient cohort, gene-expression data for the five deregulated genes in the IGCM-specific profile were correlated with the number of digitally measured immune cell numbers in the cardiac EMBs. Analysis revealed a weak correlation between Mac-1^+^ macrophages and CD45R0^+^ T memory-cells on the one hand and the computed IGCM-specific gene-profile score on the other (*p* ≤ 0.05; *r* = 0.158 and *r* = 0.161). For CD3^+^ lymphocytes, perforin^+^ cytotoxic cells, and LFA-1^+^ lymphocytes, no correlation was observed (n.s.; *r* = 0.090, *r* = 0.048 and *r* = 0.042) (see Supplemental Data Figure S2).

### 3.5. EMB-Based Diagnosis Out of The Entire Study Group

See Supplemental Table S4 for the EMB-based diagnostic findings for the entire study group.

### 3.6. Clinical and Hemodynamic Outcome of IGCM Patients at Follow-Up after Immunosuppressive Treatment

In a subgroup analysis we evaluated the response of patients with gene-profiling diagnosis of IGCM to immunosuppressive therapy. Therefore, we evaluated the observed, clinical hemodynamic outcome at follow-up (mean follow-up time: 6.4 ± 4.3 months) in patients who had received immediate immunosuppressive therapy as well as heart-failure medication after receiving an EMB-based diagnosis of IGCM. In the process, the clinical outcomes were compared between those patients whose IGCM had been histologically confirmed (*n* = 17) and those whose ICGM had been diagnosed by gene profiling (*n* = 23) (Table 4).

**Figure 4:**
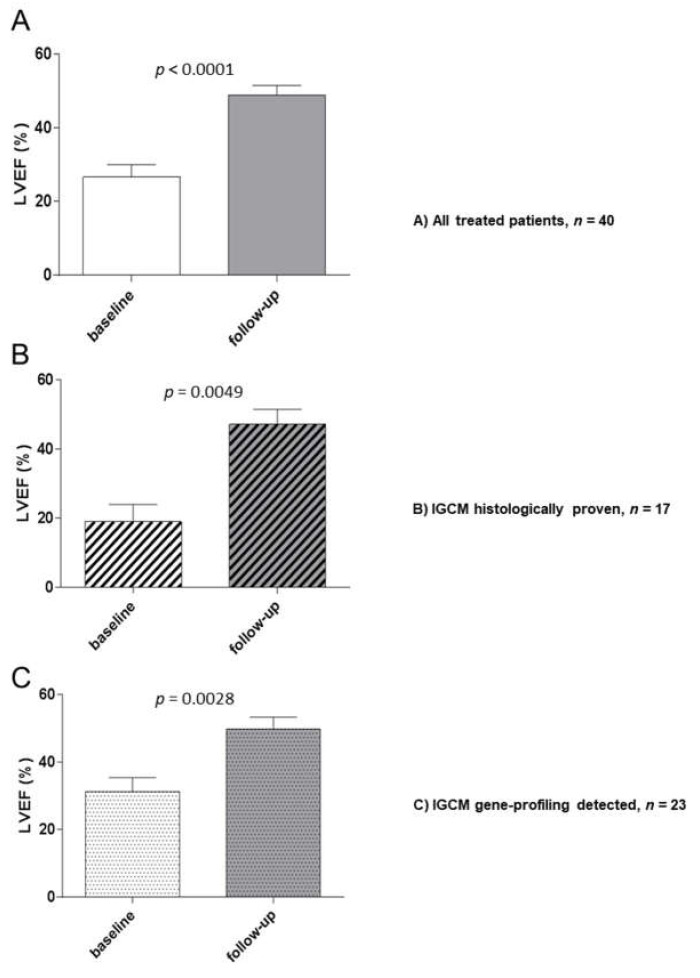
Baseline and Follow-up Hemodynamic Outcomes After Immunosuppressive Therapy for Patients with Histologically Proven or Gene-Profile-Detected IGCM. LVEF (%) was measured at baseline and during the follow-up period. (**A**) All treated patients. (**B**) Patients with histologically proven IGCM. (**C**) Patients with gene-profile-detected IGCM. The figures shown are mean values ± standard deviation; *P*-values compared to the baseline EMB are indicated.

The entire cohort of treated patients exhibited a significant improvement of LVEF (26.6 ± 15.6% to 48.9 ± 12.1%; *p* < 0.0001) following immunosuppressive treatment. Further subgroup analysis during the follow-up to immunosuppressive treatment revealed a significant improvement of LVEF (19.0 ± 14.22% to 47.25 ± 12.27%; *p* = 0.0049) in patients (*n* = 17) whose GCs had been proved by histology, i.e., through EMBs. Similarly, a significant increase of LVEF (31.3 ± 15.0% to 49.9 ± 12.4%; *p* = 0.0028) was also observed at follow-up (see Figure 4) in those cases where the patient exhibited a positive myocardial gene profile but where there was no direct histologic proof of multinuclear giant cells (*n* = 23).

**Figure 5:**
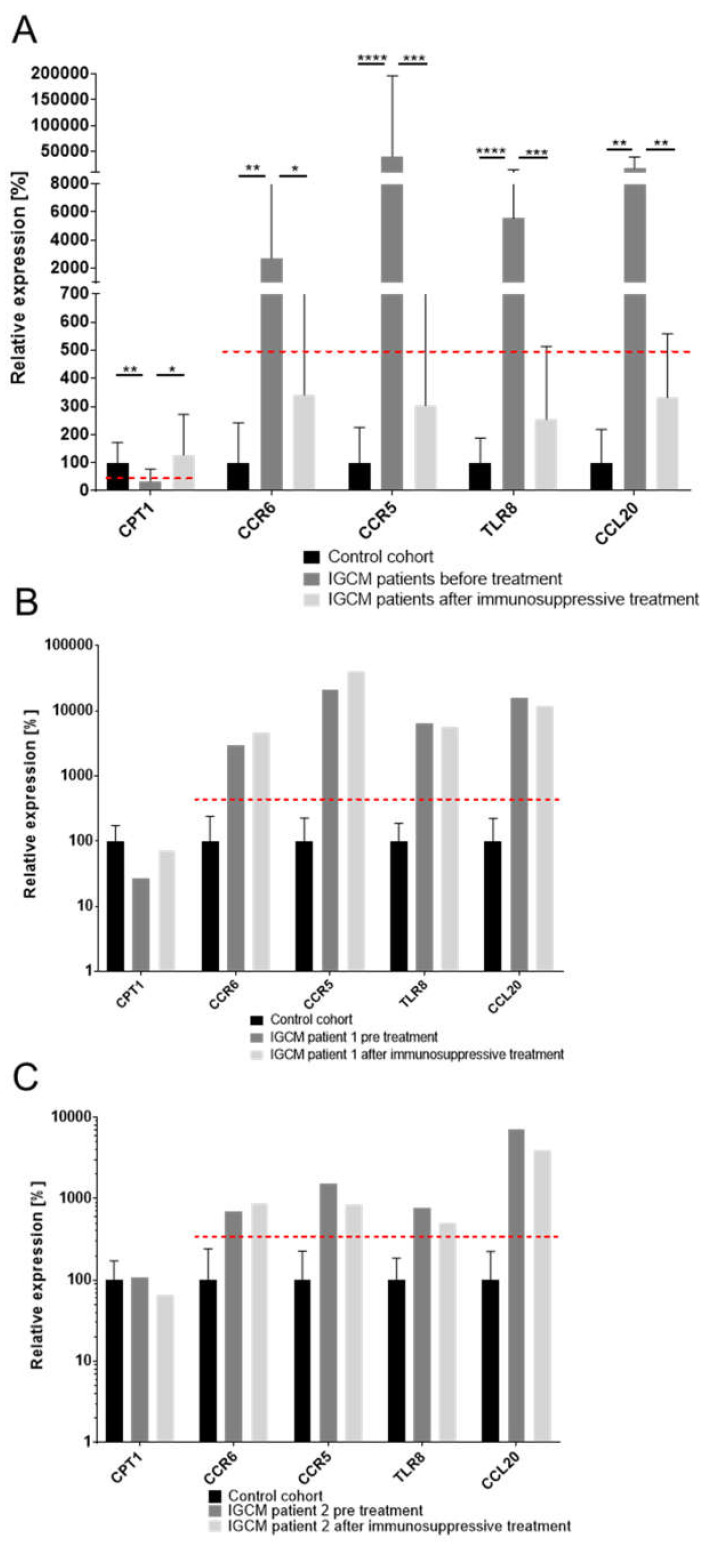
Analysis of Gene-Expression Dynamics Before and After Immunosuppressive Therapy of Patients with Histologically Proven and Gene Profile-Detected IGCM. (**A**) The gene expression of marker genes for IGCM were measured before and after applying immunosuppressive therapy and were compared to a healthy control cohort in patients with normalized gene-expression profiles at follow-up; the results indicated successful treatment. The mean expression for marker genes *CPT1*, *CCR6*, *CCR5*, *TLR8* and *CCL20* normalized at follow-up and fell below a clinically relevant threshold but did not reach the expression level of the control cohort. A dashed line indicates a clinically relevant threshold for the specific gene expression. (**B**) and (**C**) The gene expression of marker genes observed in two IGCM patients who exhibited persistent inflammation at follow-up along with a gene profile that suggested persistence of GCs after treatment. *P*-values are denoted by asterisks: * *p* ≤ 0.05, ** *p* ≤ 0.01, *** *p* ≤ 0.001, **** *p* ≤ 0.0001.

The improvement in LV function was accompanied by a reduction or complete absence of intramyocardial inflammation in the follow-up EMB (Table 4). Only two patients were found to have persistent inflammation. After extension of immunosuppressive therapy (>6 months), even these two patients became immunohistologically negative. At the time of the baseline EMB, all the patients had presented a specific pattern of deregulated genes relevant for IGCM. In the follow-up phase, the genes which had been differentially expressed in the EMBs were found to have normalized, indicating that treatment had been successful for 21 of the patients (90.0%) (Figure 5). Two patients who exhibited persistent inflammation exhibited a gene profile that was still suggestive of IGCM. Immunosuppressive treatment was therefore continued and the gene-expression values eventually normalized in accordance with clinical and histologic parameters. None of the patients died during the observation.

## 4. Discussion

The main findings of this study are: (1) Of a selected panel of 27 genes previously identified to be regulated in patients with IGCM, five genes (*CPT1, CCL20, CCR5, CCR6, TLR8*) are highly specific for patients with IGCM compared with controls; (2) Our study suggests that by using this panel of five genes, 54.3% of all IGCM cases would have been missed by using conventional histologic examination alone; (3) Patients diagnosed with IGCM using this gene profile panel respond in a similar fashion to immunosuppressive therapy as patients diagnosed with IGCM by conventional histology alone, supporting the clinical utility of gene profiling in patients presenting with unexplained acute heart failure.

In this multicenter study, myocardial gene-expression profiling to diagnose IGCM was applied in clinical practice for the first time, namely within a large cohort of patients suffering from unexplained acute cardiac decompensation. This new gene-profiling approach significantly improves the diagnosis rate for clinically suspected myocarditis and unexplained acute cardiac decompensation and helps to identify GCs which would otherwise have been missed in a purely conventional, histological examination. Our study population is unique in that it is by far one of largest group of IGCM patients ever evaluated. Fraction of IGCM-positive patients as indicated by gene profile analysis was unexpectedly high as suspected clinical diagnosis of IGCM was only assumed in 12 of 57 patients (21%) by the clinician.

Thanks to this new diagnostic test, moreover, giant-cell myocarditis is sure to be diagnosed more widely going forward. This in turn will have dramatic prognostic and therapeutic relevance for patients. After all, IGCM is the most aggressive form of active myocarditis and often has a fatal outcome. Thus, there is a high medical need to detect IGCM as early as possible so as to avoid progressive myocardial tissue damage, the eventual need for transplantation, or even cardiac death. It bears noting that, according to various multicenter studies, the five-year transplant-free survival rate is no better than 10% [3,28,29,30,31].

The only way to directly confirm the presence of giant cells is to analyze histologic sections in the form of EMBs [32], but that can be problematic [30,33]. Previous publications have postulated that immunohistologic examinations of EMBs exhibit a high sensitivity because of the diffuse inflammatory infiltration of cardiac tissue in IGCM [32]. Our study suggests, however, that there is a lack of sensitivity relying solely on conventional histopathological analysis when investigating EMB for presence of IGCM. This is where gene profiling, as a novel diagnostic tool, has several major advantages over conventional EMB analysis. For one thing, the complete biopsy can be used for nucleic acid extraction. For another, the results obtained from gene-expression profiling are more conclusive and less dependent on the operator’s experience than those obtained from optical analysis.

Although immunosuppressive therapy is considered the mainstay of medical treatment for IGCM, there is currently no consensus on how it should be executed; the optimal duration of treatment also remains undefined [22]. Moreover, the taking of additional EMBs is often needed in order to confirm a diagnosis, which can delay the initiation of treatment [22]. Also, the fulminant future course of the disease is often not foreseeable at the time the EMB is taken, so that an IGCM diagnosis is not expected. With gene-profiling, the repeated taking of EMBs could be avoided, immunosuppressive therapy could be started immediately, and optimal treatment duration could be effectively monitored.

Interestingly, we were able to show that 23 of the 26 patients with histologically proven IGCM exhibited active myocarditis according to the Dallas Classification. By contrast, active myocarditis could be observed in only *n* = 6 of the patients who presented positive gene profiles for GCs but, possibly due to the sampling error of histologic EMB analysis, presented no direct histologic proof of GCs. Moreover, the levels of lymphocytic and macrophage infiltration were significantly higher in patients who did exhibit histologic evidence of GCs than in patients who merely exhibited positive gene profiles. Normally, no GCs would have been expected in the latter cases. It follows, therefore, that the suspected diagnoses adopted by the clinicians must be viewed critically, given that they often failed to even suspect IGCM. These observations underscore the risk posed by a sampling error and the importance of new, supplementary diagnostic methods. One way to overcome these limitations and the general sampling error of purely conventional histologic examination is to apply specific gene profiling to look for indicators that multinucleated giant cells may be present in the human hearts being examined [10,11,23].

Our study was furthermore able to demonstrate, using quantitative immunohistologic staining, that the number of infiltrative cells in IGCM patients ranges widely. As expected, most patients experience a massive increase in infiltrative cells. However, our data do show that there are also patients with low inflammation and IGCM, possibly due to a focal inflammatory process. This may explain the frequently faulty suspected diagnosis made by the clinician. We consider this observation extremely important for clinicians and/or pathologists because the presence of a low inflammation in the EMB should not become an exclusion criterion for IGCM.

Another aim of the present study was to show the efficacy of immunosuppressive treatment in terms of achieving improved clinical outcomes and preventing a fatal course of IGCM [34,35,36,37,38]. Subgroup analysis of the clinical hemodynamic outcome of *n* = 40 GC patients undergoing immediate immunosuppressive therapy revealed a significant improvement of LVEF at follow-up in those patients whose GCs were proven by EMB histology. At the same time, EMB analysis revealed a significant reduction in the quantified number of inflammatory infiltrates. 

Similarly, a significant increase of LVEF after treatment was also observed in patients who exhibited positive myocardial gene profiles for GCs. EMB analysis also revealed a significant reduction in the quantified number of inflammatory infiltrates. These results underscore the clinical importance of our gene-profiling analysis, for it allows the relevant, prognostic immunosuppressive therapy to be started immediately, potentially a critical success factor. None of the patients in the overall patient cohort died during the observation period.

## 5. Conclusions

Based on 427 examined patient samples, multinucleated giant cells could be detected in only 26 patients (6.1%) through the use of histology alone. When this was supplemented by gene profiling, however, IGCM could be diagnosed in further 31 patients (7.2%), even in the absence of any direct histologic proof of giant cells. This means that 54.3% of all IGCM cases would have been missed by using conventional histologic examination alone. Which in turn highlights the importance of this new diagnostic approach. Our results show that: (1) the evaluation of EMBs is essential in successfully diagnosing IGCM, that (2) an analysis of gene-expression profiles in EMBs is of great clinical and prognostic importance when it comes to compensating for the sampling error which occurs when IGCM is diagnosed through a purely histologic examination of EMBs, and (3) patients diagnosed with IGCM using this gene profile panel respond in a similar fashion to immunosuppressive therapy as patients diagnosed with IGCM by conventional histology alone.

## 6. Limitations

This said, our study admittedly remains subject to certain caveats. For one thing, the limitations typical for retrospective cohort studies apply to our analyses. These include, among other factors, a lack of extended clinical data for all of the patients covered in this multicenter study. Furthermore, the patients in our cohorts were mainly of Caucasian ethnicity (due to the location of our study centers), which may possibly limit the applicability of our findings to other ethnic groups. On the other hand, the validity of our results tends to be corroborated by the large number of cases investigated at the core centers and by the fact that the histologic slides were independently reviewed by cardiac pathologists.

## Figures and Tables

**Table 1:** Control Group Patient Characteristics and Echocardiographical Data.

*n*	23
Age (years)	48.5 ± 12.9
LVEF (%)	51.5 ± 15.4
LVEDD (mm)	55.5 ± 9.3
TAPSE (mm)	23 ± 4.1
IVSD (mm)	11.8 ± 3.1
LVPW (mm)	11.2 ± 2.8

**Note:** LVEF = left ventricular ejection fraction; LVEDD = left ventricular end-diastolic diameter; TAPSE = tricuspid annular plane systolic excursion; IVSD = intraventricular septum diameter; LVPW = left ventricular posterior wall. The data are presented as mean ± standard deviation, and as No. (%) of subjects.

**Table 2:** Patients’ Characteristics, Hemodynamic Data and Suspected Clinical Diagnoses within the Entire Study Group.

Patient Data	Entire Cohort
*n*	427
Age (years)	47.03 ± 15.69
LVEF (%)	38.54 ± 17.89
LVEDD (mm)	54.51 ± 8.75
TAPSE (mm)	22.40 ± 5.93
IVSD (mm)	11.31 ± 2.80
LVPW (mm)	10.84 ± 2.40
NYHA I/II/III/VI (*n*)	0/0/250/177
Suspected clinical diagnoses (No., *n*):	
- AMC	180
- IGCM	30
- DCMi	100
- EOM	11
- cardiac sarcoidosis	20
- unexplained acute heart failure	86

**Note:** LVEF = left ventricular ejection fraction; LVEDD = left ventricular end-diastolic diameter; TAPSE = tricuspid annular plane systolic excursion; IVSD = intraventricular septum diameter; LVPW = left ventricular posterior wall; NYHA = New York Heart Association Classification; AMC = acute myocarditis; IGCM = idiopathic giant cell myocarditis; DCMi = dilated inflammatory cardiomyopathy; EOM = eosinophilic myocarditis; The data are presented as mean ± standard deviation, or as No. of subjects (No., *n*).

**Table 3:** Immunohistologic EMB Analysis of Intramyocardial Infiltration in GCM Patients Based on Conventional Histology or on Gene Profiling.

Patient Data	IGCM (By Histology)	IGCM (By Gene Profiling)
**Immunohistology**		
- CD3^+^ lymphocytes infiltration/mm^2^	312.4 ± 297.3	125.8 ± 196.3 *
- LFA-1^+^ lymphocytes infiltration/mm^2^	462.6 ± 413.8	183.4 ± 215.0 *
- CD45R0^+^ T memory cell infiltration/mm^2^	533.3 ± 349.6	114.9 ± 502.3 *
- perforin^+^ cell infiltration/mm^2^	16.23 ± 26.00	14.14 ± 32.23
- Mac-1^+^ macrophages infiltration/mm^2^	428.8 ± 344.0	181.1 ± 227.1 *

**Note:** Immunohistologic marker: CD3 = T-lymphocytes; LFA-1 = leukocyte function antigen-1; Mac-1 = macrophage-1 antigen; CD45R0 (UCHL1) = leucocyte common antigen; perforin = cytotoxic cells. The data are presented as mean ± standard deviation. Asterisk (*) indicates significant variance between the incidence of IGCM derived from histology and that derived from gene profiling.

**Table 4:** Clinical, Hemodynamic, and Immunohistologic EMB-analysis of Intramyocardial Infiltration in GCM patients (*n* = 40) Based on Conventional histology and on Gene Profiling at Baseline and After Immunosuppressive Therapy.

Patient Data	IGCM (By Histology) *At baseline*	IGCM (By Histology) *After therapy*	IGCM (By Gene Profiling) *At baseline*	IGCM (By Gene Profiling) *After therapy*
*n*	17	17	23	23
LVEF (%)	19.0 ± 14.22 *	47.25 ± 12.27	31.3 ± 15.0 *	49.9 ± 12.4
LVEDD (mm)	56.23 ± 5.23	55.62 ± 8.43	56.43 ± 7.28	55.93 ± 4.29
TAPSE (mm)	20.48 ± 5.13	21.23 ± 5.34	21.96 ± 7.53	21.81 ± 6.33
IVSD (mm)	10.98 ± 3.15	10.42 ± 4.21	11.12 ± 3.15	11.02 ± 4.12
LVPW (mm)	10.14 ± 2.41	10.05 ± 2.24	10.25 ± 2.16	10.58 ± 2.07
NYHA I/II/III/VI	0/0/6/11	0/11/6/0	0/0/11/12	0/18/5/0
Immunohistologic Analysis				
CD3^+^ T lymphocytes infiltration/mm^2^	397.3.4 ± 305.3 **	23.57 ± 19.23	169.1 ± 111.5 **	15.72 ± 17.94
LFA-1^+^ lymphocytes infiltration/mm^2^	612.6 ± 405.5 **	40.91 ± 21.00	190.8 ± 119.6 *	23.77 ± 20.69
CD45R0^+^ T memory cells/mm^2^	584.5 ± 340.5 *	63.95 ± 59.47	208.1 ± 158.3	33.99 ± 25.62
perforin^+^ cytotoxic cells/mm^2^	21.70 ± 28.42	0.97 ± 0.72	39.22 ± 48.64	2.17 ± 4.73
Mac-1^+^ macrophages infiltration/mm^2^	569.3 ± 311.5 **	59.44 ± 18.92	200.5 ± 111.3 **	40.10 ± 26.42

**Note:** LVEF = left ventricular ejection fraction; LVEDD = left ventricular end-diastolic diameter; TAPSE = tricuspid annular plane systolic excursion; IVSD = intraventricular septum diameter; LVPW = left ventricular posterior wall; NYHA = New York Heart Association Classification. Immunohistologic marker: CD3 = T-lymphocytes, LFA-1 = leukocyte function antigen-1, Mac-1 = macrophage-1 antigen, CD45R0 (UCHL1) = leucocyte common antigen, perforin = cytotoxic cells. The data are presented as mean ± standard deviation, and as No. of subjects. Significant variance between the value at baseline and after therapy are indicated (* *p* ≤ 0.05; ** *p* ≤ 0.01).

## Supplementary Materials

The following are available online at https://www.mdpi.com/2077-0383/9/9/2689/s1, Table S1: Detailed Endomyocardial Results in Control Group (*n* = 23); Table S2: Potential marker genes for gene profile development; Figure S1: Gene profile performance analysis for IGCM detection; Table S3: ROC of gene profile performance analysis for IGCM detection; Table S4: EMB-Based Diagnostic Findings for the Entire Study Group; Figure S2: Correlation of IGCM-specific gene-expression profile score with the number of digitally measured immune cells in EMBs in the total patient cohort.

## Abbreviations

CCL20	chemokine (C-C motif) ligand 20
CCR5	chemokine receptor 5
CCR6	chemokine receptor 6
CPT1	carnitine palmitoyltransferase I
DCM	dilated cardiomyopathy
DCMi	inflammatory DCM
EMB	endomyocardial biopsy
EOM	eosinophilic myocarditis
EvG	elastic van Gieson stain
GCs	giant cells
IGCM	idiopathic giant cell myocarditis
IVSD	intraventricular septum diameter
LVEDD	left ventricular end-diastolic diameter
LVEF	left ventricular ejection fraction
LVPW	left ventricular posterior wall
NYHA	New York Heart Association Classification
RT-qPCR	reverse transcription-quantitative polymerase chain reaction
TAPSE	tricuspid annular plane systolic excursion
TLR8	toll-like receptor 8
