# Supplementary material for: Evaluation of Myocardial Gene Expression Profiling for Superior Diagnosis of Idiopathic Giant-Cell Myocarditis and Clinical Feasibility in a Large Cohort of Patients with Acute Cardiac Decompensation
Source: J Clin Med. 2020 Aug 19;9(9):2689. doi: 10.3390/jcm9092689 (PMC7563288; doi:10.3390/jcm9092689)
Supplement: Supplementary file 1 [file jcm-09-02689-s001.pdf]

Control group

23 age and gender-matched retrospectively searched out patients without EMB-proven intramyocardial inflammation/viral infection served as a peer group to create the control-group profiling.

Table S1. Detailed Endomyocardial Results in Control Group (*n* = 23)

Histological findings	All
- Myocytolysis, *n*	0
- Giant cells, *n*	0
- Eosinophils, *n*	0
- Myocyte diameter, µm	21.3 ± 0

Immunohistological findings	All
- $CD3^+$ lymphocytes infiltration/mm^2	3.1 ± 2.6
- LFA-1^+ lymphocytes infiltration/mm^2	7.8 ± 4.3
- $CD45R0^+$ T memory cell<br>- infiltration/mm^2	12.2 ± 8.0
- $perforin^+$ cell<br>- infiltration/mm^2	0.3 ± 0.6
- Mac-1^+ macrophages<br>- infiltration/mm^2	18.6 ± 7.5

Note: CD3 = T lymphocytes; LFA-1 = lymphocyte function-associated antigen-1; CD45R0 (UCHL1) = leucocyte common antigen; perforin = cytotoxic cells; Mac-1 = macrophage-1 antigen.

Table S2. Potential marker genes for gene profile development.

Abbreviation	Gene name	Synonym	RefSeq mRNA ID	RefSeq Protein ID	Uniprot ID	Entrez Gene ID	Reference	Molecular function
A4GALT	Lactosylceramide 4-alpha-galactosyltransferase	P(k)	NM_017436	NP_059132	Q9NPC4	53947	Lassner et al., 2014 Kuehl et al., 2013	M
ATP5I	ATP synthase subunit g		NM_007100	NP_009031	P56385	521	Kuehl et al., 2013	EM, ce
B3GALT5	Beta-1,3-galactosyltransferase 5	GLCT5	NM_033172	NP_149362	Q9Y2C3	10317	Kuehl et al., 2013	M

BAG5	BAG family molecular chaperone regulator 5		NM_004873	NP_004864	Q9UL15	9529	Kuehl et al., 2013	CC
CCL20	CC-chemokine ligand 20	ST38	NM_004591	NP_004582	P78556	6364	Lassner et al., 2014 Kuehl et al., 2013	IR
CCR5	C-C chemokine receptor type 5	IDDM22	NM_001100168	NP_001093638	P51681	1234	Lassner et al., 2014	IR
CCR6	C-C chemokine receptor type 6	STRL22	NM_031409	NP_113597	P51684	1235	Lassner et al., 2014	IR
CDKN1A	cyclin-dependent kinase inhibitor	WAF1	NM_078467	NP_510867	P38936	1026	Kuehl et al., 2013	CC
CPT1A	Carnitine palmitoyltransferase I	L-CPT1	NM_001876	NP_001867	P50416	1374	Lassner et al., 2014 Kuehl et al., 2013	M, ce
DHODH	Dihydroorotate dehydrogenase		NM_001361	NP_001352	Q02127	1723	Lassner et al., 2014 Kuehl et al., 2013	EM, mt
FOXP3	Forkhead box P3	XPID	NM_014009	NP_054728	Q9BZS1	50943	Lassner et al., 2014 Kuehl et al., 2013	IR
GBGT1	Globoside alpha-1,3-N-acetylgalactosaminyltransferase 1	UDP-GalNAc	NM_021996	NP_068836	Q8N5D6	26301	Kuehl et al., 2013	M
IFNB1	Interferon beta 1	IFNB	NM_002176	NP_002167	P01574	3456	Kuehl et al., 2013	IR
IL10	Interleukin 10	IL10A	NM_000572	NP_000563	P22301	3586	Lassner et al., 2014 Kuehl et al., 2013	IR
IL17D	Interleukin 17D	IL-17D	NM_138284	NP_612141	Q8TAD2	53342	Lassner et al., 2014	IR
IL6R	Interleukin 6 receptor	CD126	NM_181359	NP_852004	P08887	3570	Lassner et al., 2014 Kuehl et al., 2013	IR
MT-CYB	Cytochrome b	UQCR3		YP_003024038	P00156	4519	Kuehl et al., 2013	EM, mt
MT-ND1	NADH ubiquinone oxidoreductase core subunit 1	NAD1		YP_003024026	P03886	4535	Lassner et al., 2014	EM, mt

MT-ND4	NADH ubiquinone oxidoreductase core subunit 4	ND4		YP_0030240 35	P03905	4538	Lassner et al., 2014	EM, mt
NDUFS1	NADH-ubiquinone oxidoreductase 75 kDa subunit	CI-75k	NM_005006	NP_004997	P28331	4719	Kuehl et al., 2013	EM, ce
POLG	DNA polymerase subunit gamma	POLGA	NM_002693	NP_002684	P54098	5428	Kuehl et al., 2013	EM, ce
SLC25A5	ADP/ATP translocase 2	T3	NM_001152	NP_001143	P05141	292	Kuehl et al., 2013	EM, ce
SOD2	Superoxide dismutase 2		NM_001322 817	NP_0013097 49	P04179	6648	Kuehl et al., 2013	EM, ce
TLR8	Toll-like receptor 8	CD288	NM_138636	NP_619542	Q9NR97	51311	Lassner et al., 2014	IR
TLR9	Toll-like receptor 9	CD289	NM_017442	NP_059138	Q9NR96	54106	Lassner et al., 2014	IR
TNF	Tumor necrosis factor	TNFSF2	NM_000594	NP_000585	P01375	7124	Lassner et al., 2014	IR
UQCR	Ubiquinol-cytochrome c reductase	UQCR	NM_006830	NP_006821	O14957	10975	Lassner et al., 2014	EM,ce

Note: M = metabolism; EM = energy metabolism; ce = chromosomally encoded gene; CC = cell cycle regulation; IR = immune response; Major molecular function and chromosomal (cs) or mitochondrial (mt) genome localization of mitochondrial genes are indicated.

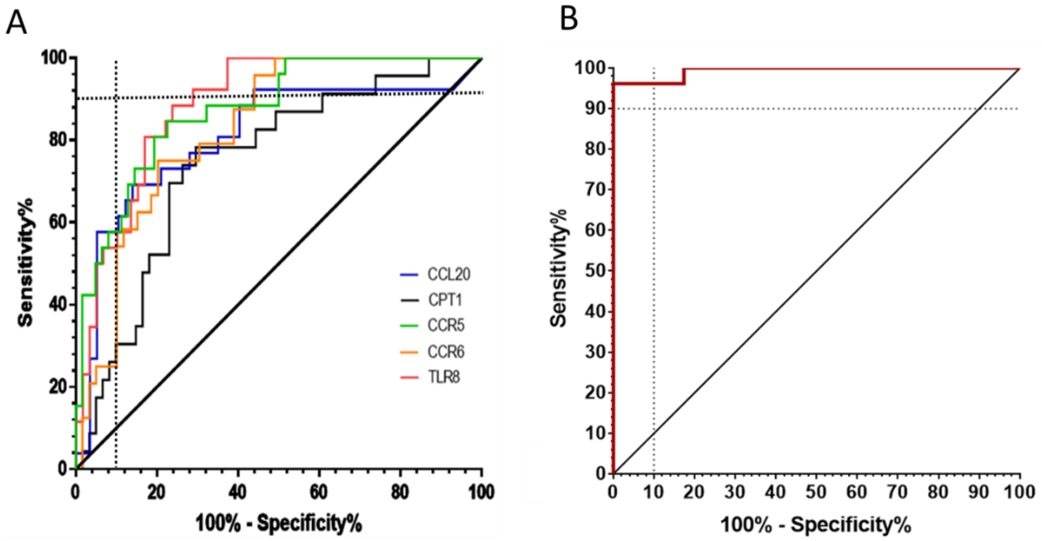

Figure S1. Gene profile performance analysis for IGCM detection. (A) Single gene profile performance analysis of each marker gene to detect IGCM and (B) gene profile performance of final gene profile is demonstrated by ROC. Solid black line represents chance level. Dotted lines indicate 90 % specificity and 90 % sensitivity threshold levels. Detailed information on single gene performance and integrated profile for detection of IGCM are given in Table S3.

Table S3. ROC of gene profile performance analysis for IGCM detection.

Genes	AUC	SE	95% CI
CCL20	0.807	0.05739	0.6945 to 0.9195
CCR5	0.8722	0.03909	0.7956 to 0.9488
CCR6	0.8249	0.04538	0.7359 to 0.9138

CPT1	0.7463	0.05867	0.6313 to 0.8612
TLR8	0.8866	0.03466	0.8186 to 0.9545
Final gene profile	0.9933	0.00782	0.978 to 1.009

Table S4. EMB-Based Diagnostic Findings for the Entire Study Group.

Patient Category	Total
Entire Cohort (*n*)	427
IGCM	57
AMC	40
DCMi	194
EOM	4
Cardiac sarcoidosis	5
DCM	147

Viral PCR Analysis of DCMi Patients.

Patient Category	Total
Entire Cohort (*n*)	194
Coxsackievirus	0
Enterovirus	1
Human Herpes Virus 6	24
Epstein-Barr Virus	4
Erythrovirus	78

Note: IGCM = idiopathic giant cell myocarditis; AMC = acute myocarditis; DCMi = dilated inflammatory cardiomyopathy; EOM = eosinophilic myocarditis; DCMi = inflammatory dilated cardiomyopathy. The data are presented as No. of subjects.

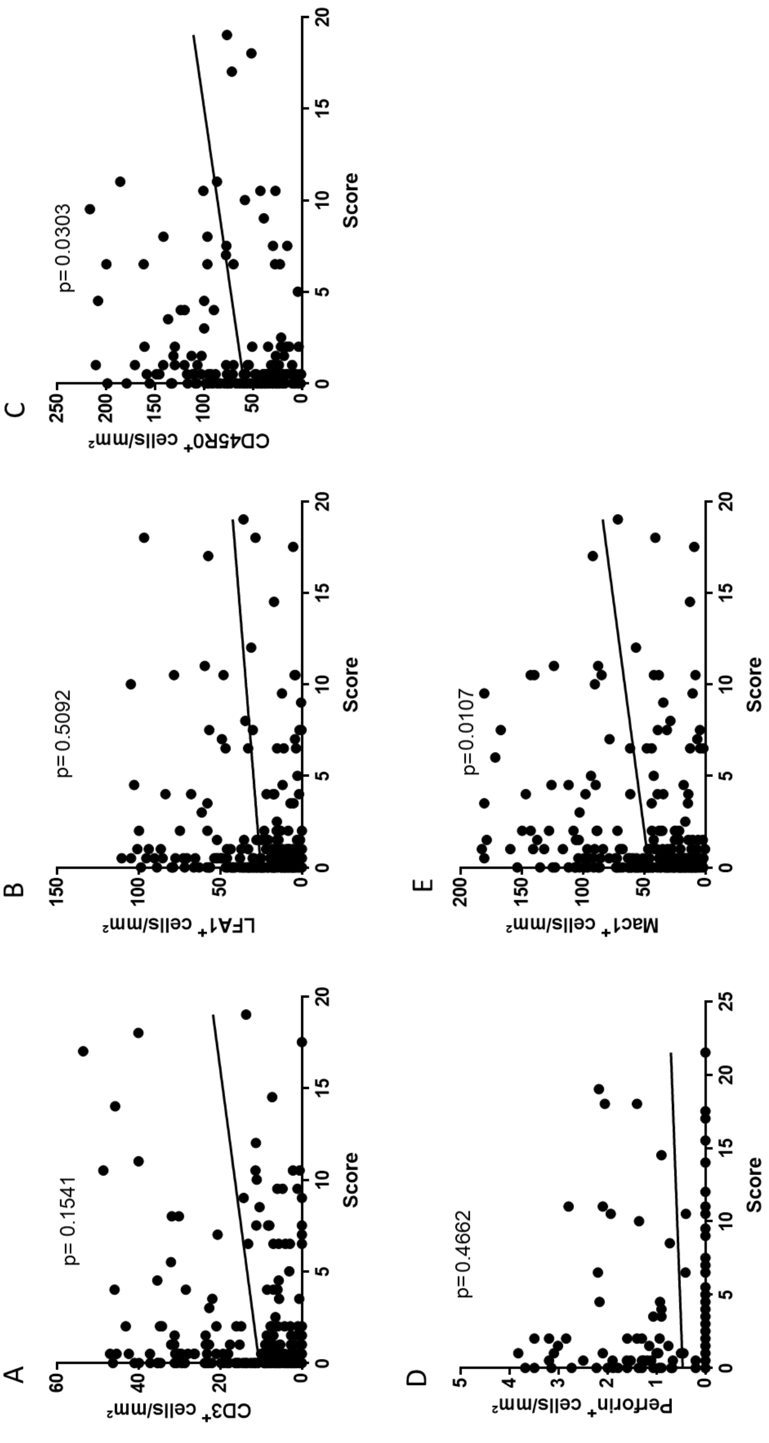

Figure S2 Correlation of IGCM-specific gene-expression profile score with the number of digitally measured immune cells in EMBs in the total patient cohort. Correlation of (A) CD3+, (B) LFA-1+, (C) CD45+, (D) perforin+, and (E) Mac-1+-cells with computed gene-profiling score is shown. **Note:** Immunohistological marker: CD3 = T-lymphocytes, LFA-1 = leukocyte function antigen-1, Mac-1 = macrophage-1 antigen, CD45R0 (UCHL1) = leucocyte common antigen, perforin = cytotoxic cells. *P*-values are indicated.
